# Polymorphism, selection and tandem duplication of transferrin genes in Atlantic cod (*Gadus morhua*) - Conserved synteny between fish monolobal and tetrapod bilobal transferrin loci

**DOI:** 10.1186/1471-2156-12-51

**Published:** 2011-05-25

**Authors:** Øivind Andersen, Maria Cristina De Rosa, Davide Pirolli, Ave Tooming-Klunderud, Petra E Petersen, Carl André

**Affiliations:** 1 Nofima Marin, P. O. Box 5010, N-1430 Ås, Norway; 2 Norwegian University of Life Sciences, P. O. Box 5003, N-1430 Ås, Norway; 3 Institute of Chemistry of Molecular Recognition - CNR and Institute of Biochemistry and Clinical Biochemistry, Catholic University of Rome, 00168 Rome, Italy; 4 Centre for Ecological and Evolutionary Synthesis (CEES), Department of Biology, University of Oslo, P.O. Box 1066 Blindern, N-0316 Oslo, Norway; 5 Aquaculture Research Station of the Faroes, FO-430 Hvalvík, Faroe Islands; 6 Department of Marine Ecology-Tjärnö, Gothenburg University, S-45296 Strömstad, Sweden

**Keywords:** Monolobal transferrin, Atlantic cod, tandem duplication, adaptation, positive selection, molecular modeling

## Abstract

**Background:**

The two homologous iron-binding lobes of transferrins are thought to have evolved by gene duplication of an ancestral monolobal form, but any conserved synteny between bilobal and monolobal transferrin loci remains unexplored. The important role played by transferrin in the resistance to invading pathogens makes this polymorphic gene a highly valuable candidate for studying adaptive divergence among local populations.

**Results:**

The Atlantic cod genome was shown to harbour two tandem duplicated serum transferrin genes (*Tf1*, *Tf2*), a melanotransferrin gene (*MTf*), and a monolobal transferrin gene (*Omp*). Whereas *Tf1 *and *Tf2 *were differentially expressed in liver and brain, the *Omp *transcript was restricted to the otoliths. Fish, chicken and mammals showed highly conserved syntenic regions in which monolobal and bilobal transferrins reside, but contrasting with tetrapods, the fish transferrin genes are positioned on three different linkage groups. Sequence alignment of cod *Tf1 *cDNAs from Northeast (NE) and Northwest (NW) Atlantic populations revealed 22 single nucleotide polymorphisms (SNP) causing the replacement of 16 amino acids, including eight surface residues revealed by the modelled 3D-structures, that might influence the binding of pathogens for removal of iron. SNP analysis of a total of 375 individuals from 14 trans-Atlantic populations showed that the *Tf1*-NE variant was almost fixed in the Baltic cod and predominated in the other NE Atlantic populations, whereas the NW Atlantic populations were more heterozygous and showed high frequencies of the *Tf-*NW SNP alleles.

**Conclusions:**

The highly conserved synteny between fish and tetrapod transferrin loci infers that the fusion of tandem duplicated *Omp*-like genes gave rise to the modern transferrins. The multiple nonsynonymous substitutions in cod Tf1 with putative structural effects, together with highly divergent allele frequencies among different cod populations, strongly suggest evidence for positive selection and local adaptation in trans-Atlantic cod populations.

## Background

There is an increasing interest in studying genotype by environment interactions in terrestrial and aquatic organisms to clarify how different populations might have diverged to become adapted to local habitats, and to understand how populations may respond to human-induced selection. In marine fishes the population structure is expected to be weak because of extensive gene flow, and it might be questioned whether adaptive responses to changes in physico-chemical conditions or selective harvesting have occurred in this large vertebrate group. These objections have been challenged by an increasing number of genetic studies of population structure [1-3]. The focus has recently changed from the rather descriptive population genetic analyses to an intensive search for functional genes subjected to natural selection [4,5]. The limited number of fitness-related genes reported to be associated with adaptive traits in marine or euryhaline fishes includes the three-spined stickleback ectodysplasin [6], European flounder Hsc70 [7], killifish lactate dehydrogenase [8], and Atlantic cod pantophysin [9,10] and hemoglobin [11]. The latter study provided evidence for local adaptation in cod populations by identifying molecular mechanisms underlying the different oxygen binding properties of the cod hemoglobins in temperate and Arctic waters. An alternative strategy for identifying adaptive population divergence is to scan transcriptome databases, or the whole genome when available, for polymorphic loci to be analysed in different populations [5]. Evidence of directional selection in Atlantic cod was provided by genotyping multiple gene-associated single nucleotide polymorphisms (SNPs) in Northeast and Northwest Atlantic populations [12-14]. Outlier loci were shown to be correlated with temperatures and/or salinity conditions that might be associated with local adaptation. However, documentation of molecular changes underlying adaptive traits requires additional information about the specific genes involved and the regulatory or structural implications of the identified mutations.

Iron is an essential element required for the growth and survival of most organisms, and the iron balance is therefore tightly regulated by several interacting iron-binding factors [15]. Serum transferrin plays a crucial role in iron metabolism as it provides most of the iron required for organismal functions [16]. The two homologous iron-binding lobes of modern transferrins are believed to have evolved by gene duplication of a primitive monolobal form [17,18], which has been reported in a surprisingly few species. Most insect transferrins bind only one ferric iron, but this is because of extensive deletions in the C-terminal lobe [19]. Evidence of a 44-kDa hagfish transferrin with only one iron-binding site was contradicted in a later study [20], and a report on monolobal rat hemiferrin turned out to be erroneous [21]. True monolobal transferrin has therefore been claimed to be present only in ascidian species of the Urochordates [22-24]. A transferrin-like otolith matrix protein (OMP) with a single potential metal binding site was identified in rainbow trout and zebrafish [25-27], but the protein was proposed to represent a partial sequence of the membrane-bound melanotransferrin (MTf) [28]. The possibility for an alternatively spliced monolobal variant of the *MTf *gene might be excluded by the identification of both genes in the fish genome.

By limiting the availability of iron for replicating pathogens transferrin provides resistance to bacterial infections [29,30], but an iron-independent role of transferrin in the immune system is evidenced by the involvement of transferrin and its receptor in early T cell differentiation in the thymus [31]. The conserved dual functions of transferrin in the iron metabolism and immune response were recently demonstrated in sea bass, which responded to both bacterial infection and altered iron status by modulating the liver and brain expression of transferrin [32]. Bacterial species have a variety of mechanisms for obtaining transferrin-bound iron [33], and competition for iron from bacterial pathogens could potentially be a strong source of natural selection on vertebrate transferrins. For example, Newton et al. [34] provided evidence for a transferrin allele as a host-level risk factor in naturally occurring equine respiratory disease, and Jurecka et al. [35] showed that particular transferrin alleles were associated with the resistance of common carp to the blood parasite *Trypanoplasma borrelli *probably as a direct effect of binding to the transferrin receptor of the parasite.

Atlantic cod has been one of the major fish resources on the continental shelf and banks on both sides of the North Atlantic Ocean, and has undergone a complex pattern of phylogenetic evolution, including population fluctuations attributable to long-term geological events, short-term ecological history and contemporary antropogenic fishing and environmental shifts [36,37]. Serum transferrin polymorphism in Atlantic cod was reported almost 50 years ago [38], and the presence of two distinct cod populations at the Faroe Islands was proposed by analysing the frequencies of five transferrin types [39]. The important role played by transferrin in the immune response makes this polymorphic gene highly valuable for identifying adaptive divergence between local populations. Here we report on selective signatures of a tandem duplicated transferrin gene in trans-Atlantic populations, and provide novel evidence for the evolution of modern transferrins from a monolobal transferrin locus.

## Results

### Four cod transferrin genes

**Figure 1:**
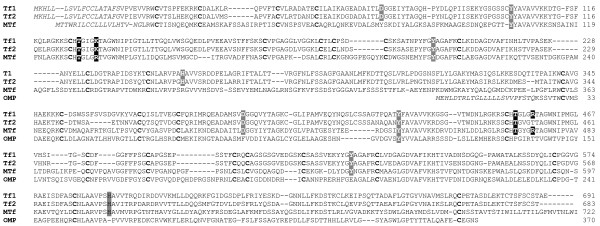
**Sequence alignment of cod Tf1, Tf2, MTf and OMP**. Hyphens are introduced to optimize the alignment. Conserved Cys residues (bold) and residues for binding iron (grey) and carbonate anion (black) are indicated. Putative signal peptide is shown in italics.

**Figure 2:**
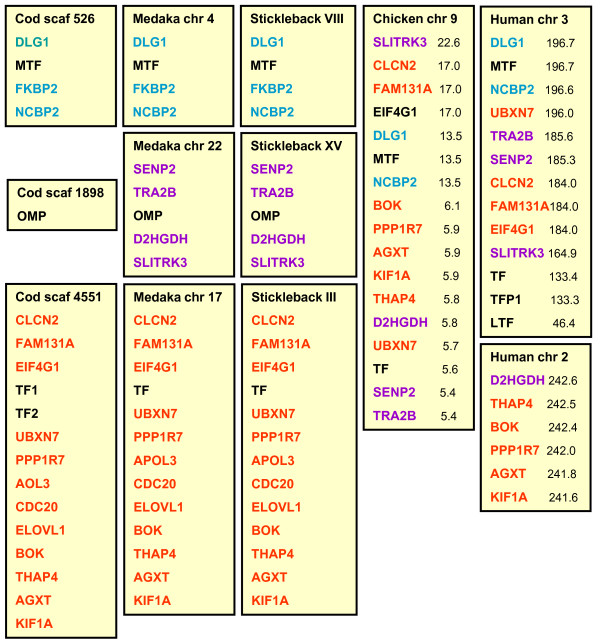
**Conserved synteny between fish, chicken and human transferrin loci**. The flanking genes are color-coded to visualize their linkage to Tf, Omp and MTf in fish. Chromosomal positions of the chicken and human genes are indicated.

Two full-length Atlantic cod *Tf *cDNAs with different 3'UTR length were identified in the GAFFA database of the Norwegian coastal cod population. The open reading frame (ORF) of 2073 nucleotides (nt) was identical in the two cDNAs, and the 689 amino acids (aa) of the designated cod Tf1 were predicted (Figure 1). The corresponding *Tf1 *gene spanned about 8 kb of scaffold GmG100427sc4451 in the reference genome of Atlantic cod representing the Northeast Arctic population (Figure 2). Aligning the *Tf1 *genomic and cDNA sequences revealed 17 exons sharing 100% sequence identity with the *Tf1 *cDNA. The allelic variant of cod *Tf1 *identified in the two Northeast Atlantic populations was named *Tf1*-NE.

**Figure 3:**
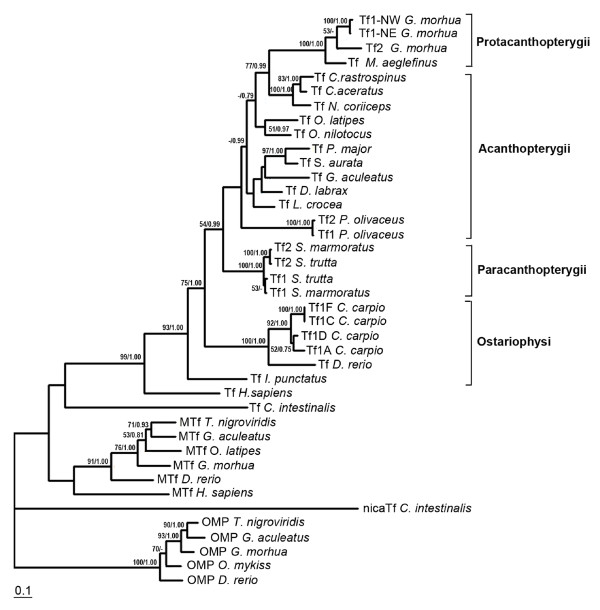
**Phylogenetic tree of chordate transferrins**. The Maximum Likelihood tree is shown. Numbers at the nodes represent Maximum Likelihood and Bayesian support values, respectively. Only values above 50/0.75 are shown.

**Figure 4:**
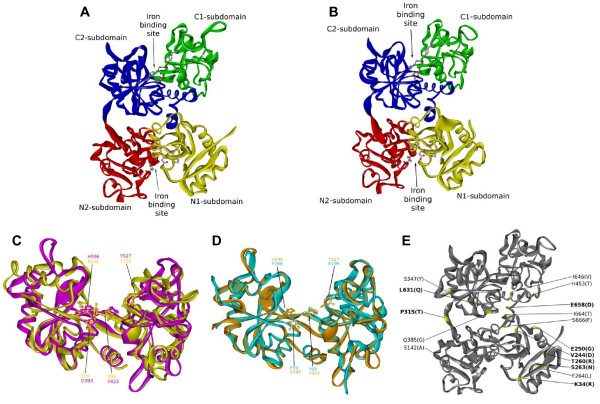
**Ribbon representation of the calculated 3D models of the Atlantic cod transferrin forms**. **A**. Cod Tf1 (NE variant) displaying the iron-binding N- and C-lobes each consisting of two subdomains. The conserved iron-binding residues are shown in ball and stick. **B**. Cod Tf2. **C**. Superimposition between the N- (yellow) and C-lobe (pink) of cod MTf. The C-lobe contains the four iron-binding residues Asp393, Tyr423, Tyr527 and His596, while the N-lobe is lacking the crucial Asp and His residues. **D**. Superimposition between cod OMP (green) and the C-lobe (brown) of cod MTf visualizing the monolobal structure of OMP, which are lacking three of the four potential iron-binding residues. **E**. NW variant of cod Tf1 showing the location of the 16 substituted amino acids, including eight surface residues (bold).

**Figure 5:**
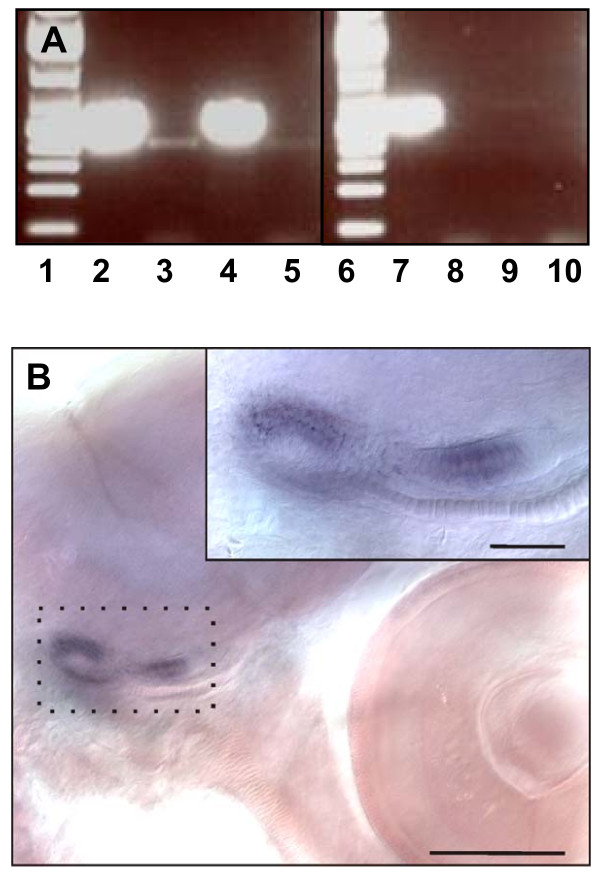
**Gene expression of Atlantic cod *Tf1*, *Tf2 *and *Omp***. **A**. RT-PCR analysis of cod *Tf1 *and *Tf2 *expression. MW marker (lane 1, 6), liver *Tf1 *(lane 2, 7), liver *Tf2 *(lane 3, 8), brain *Tf1 *(lane 4, 9), brain *Tf2 *(lane 5, 10). Lane 2-5; 35 cycles, lane 7-10; 25 cycles. **B**. WISH staining of cod *Omp *in the larval otoliths. Scale bars represent 200 μm (main image) and 50 μm (insert).

A second transferrin gene designated cod *Tf2 *was found about 16.5 kb downstream of *Tf1 *(Figure 2). The 17 exons of *Tf2 *were identified by aligning the tandem duplicated *Tf *genes, and the predicted 683 aa of Tf2 shared 84% identity with cod Tf1 (Figure 1). The two serum transferrins showed 82 and 78% identity, respectively, with haddock Tf, but only 53-63% identity with the non-gadoid fish Tfs presented in the constructed phylogenetic tree (Figure 3). Although the gadoids clustered with the Antarctic notothenioids examined, the phylogenetic tree seems to be consistent with the taxonomic classification of teleosts, while man and *Ciona *formed separate branches. Four potential iron-binding residues forming a DYYH motif [40] are conserved in both the N- and C-lobe of cod Tf1 and Tf2, and the two lobes contained 12 and 14 conserved Cys residues, respectively, involved in the formation of disulphide bridges (Figure 1). However, the basic Arg residue serving as the principal anchor for the synergistic carbonate anion has been changed to Lys131 in the N-lobe of Tf1 and to Thr455 in the C-lobe of Tf2. The corresponding Arg124Lys mutation in the N-lobal anion binding site of human Tf generated a protein much more facile in releasing iron [41]. The 3D structure of Tf1 and Tf2 illustrates the bilobal nature of the proteins in which each lobe is divided into two subdomains connected by a hinge that give rise to a deep cleft containing the iron-binding residues (Figure 4A, B). The expression of the two genes was semi-quantified by RT-PCR using gene specific primers. The transcripts of both *Tf1 *and *Tf2 *were identified in the liver and brain, but *Tf2 *was expressed at very low levels compared to *Tf1 *(Figure 5A). Reducing the number of PCR cycles from 35 to 25 diminished the *Tf1 *amplicon considerably only in the brain, indicating abundant expression of *Tf1 *in the liver.

A cod melanotransferrin (*MTf*) gene and its cDNA were identified in scaffold GmG100427s526 of the reference genome (Figure 2) and in the GAFFA database, respectively. Sequence alignment revealed 17 exons, and the ORF of 2166 nt encodes the predicted 722 aa of cod MTf, which showed only 36% identity with cod Tf1 and Tf2 (Figure 1). In comparison, cod MTf shared 62-69% identity with the MTf of stickleback, medaka, pufferfish and zebrafish. The cluster of fish and human MTf in the phylogenetic tree was, however, not supported by the low boot strap value (<50%), and *Ciona *Tf formed a separate branch (Figure 3). All four iron-binding residues (Asp393, Tyr423, Tyr527, His596) are present in the C-lobe of cod MTf, whereas Tyr56 and Arg257 in the N-lobe have replaced the crucial Asp and His residues, respectively (Figure 4C). Thus, the iron-binding activity is apparently intact only in the C-lobe of cod MTf, in contrast to the binding of iron to the N-lobe of mammalian MTf, in which the C-lobe is involved in membrane anchoring [42]. Although the residues for iron binding are not conserved in MTf, the overall sequence identity between the two lobes in MTf is higher than those of Tf in both man (48% and 42%, respectively) and cod (42% and 36%; Tf1, 38%; Tf2). Consistently, the N- and C-lobes of cod MTf aligned closely in the superimposition of the two lobes (Figure 4C), and a root mean square deviation (RMSD) of 1.5 Å over 264 aligned C-α atoms was calculated.

A monolobal transferrin of only 370 aa was predicted from the 9 exons identified in a gene spanning the entire scaffold GmG100427s1898 of 37.8 kb in the reference cod genome (Figure 1 and 2). The completeness of the gene was confirmed by the identification of the orthologous gene and the conserved flanking genes *Senp2*, *Tra2b*, *D2hgdh *and *Slitrk3 *in the other fish genomes available, including medaka and stickleback (Figure 2). Whole mount *in situ *hybridization revealed the exclusive staining of the otoliths in the cod larvae (Figure 5B), and the predicted protein showed 79 and 76% identity, respectively, with the otolith matrix protein (OMP) of rainbow trout and zebrafish [25,27]. Cod OMP shared low identity with the N- and C-lobes, respectively, of Tf1 (32% and 31%), Tf2 (34% and 32%) and MTf (38% and 35%), which are slightly higher than the identities of 31 and 30% between the monolobal nicaTf and the N- and C- lobes, respectively, of Tf in *Ciona intestinalis *[43]. The monolobal nature of cod OMP was illustrated by the 3D model of the protein superimposed on the C-lobe of cod MTf (Figure 4D). The C-α RMSD of 1.52 and 0.60 Å, respectively, between OMP and the N- and C-lobes of MTf (over 271 and 315 aligned C-α atoms, respectively) indicates a major structural similarity of OMP to the C-lobe, although cod OMP is lacking all the key residues for iron and carbonate anion binding, except for the conserved Tyr111 (Figure 1).

### Cod Tf1 polymorphisms

Polymorphisms in the cod *Tf1 *were found by comparing the *Tf1*-NE variant with a *Tf *cDNA isolated from a Northwest (NW) Atlantic cod population [44]. The missing N-terminal coding sequence of the latter variant named *Tf1*-NW was PCR amplified from a heterozygous Faroe cod, and a total of 22 SNPs were identified by aligning the protein coding sequences of the two cod *Tf1 *variants (Additional file 1; Figure S1). The 18 non-synonymous substitution sites were found to cause 16 amino acid replacements, including eight surface residues (Figure 4E). Additionally, E160 is deleted in the NW variant probably as the result of alternative splicing of exons 5 and 6. The 3D model of the NE and NW variants revealed no structural differences in the iron-binding sites, and the high structural similarity between the two variants was expressed by the calculated RMSD of only 0.64Å between Cα atoms. The *d*_*N*_/*d*_*S *_ratio of 5.8 between the NW and NE variants is significantly greater than 1 (*p *= 0.0013) indicating strong positive selection on the cod *Tf1 *gene.

### Population genetic analyses

**Figure 6:**
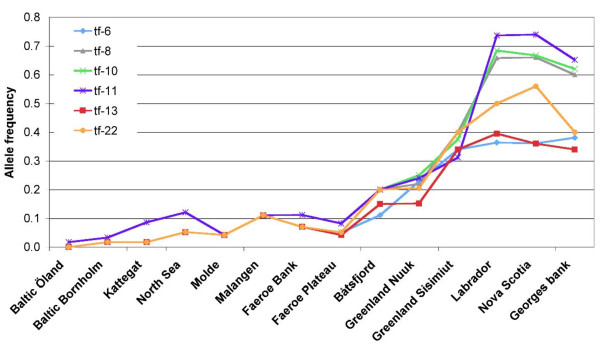
**Allele frequencies of six SNP loci spanning the *Tf1 *gene in cod samples collected across the North Atlantic**. See Table 1 and Additional file 2; Figure S2 for sampling locations, and Additional file 3: Table S1 for SNP alleles.

**Figure 7:**
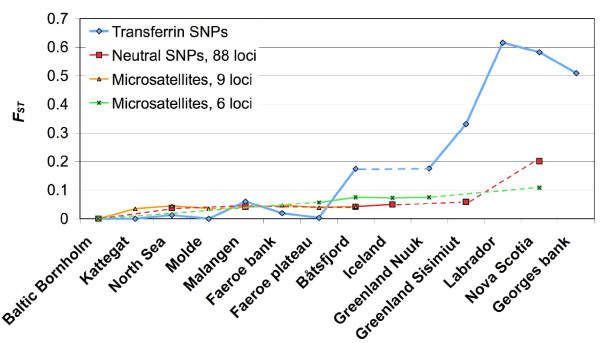
**Population differentiation in the *Tf1 *gene compared with neutral genetic markers**. Population pair-wise *F*_ST _values between Baltic cod and other Atlantic cod samples, following a transect from east to west, for the six *Tf1 *SNPs combined and for published estimates of differentiation in genetically neutral SNP and microsatellite loci (see text for details).

The cod *Tf1 *polymorphisms were investigated in 14 cod populations covering the North Atlantic (Table 1, Additional file 2; Figure S2) by genotyping six of the 22 identified SNPs. The NE haplotype was almost fixed in the Baltic cod samples and predominated in the other eastern Atlantic samples, although at slightly different frequencies in the Northeast Arctic cod and the Greenland Nuuk sample (Figure 6, Additional file 3: Table S1). In contrast, the NW alleles of the three SNPs tf-8, tf-10 and tf-11 dominated in the Canadian samples examined, whereas alleles of the N-terminal (tf-6) and the C-terminal SNPs (tf-13, tf-22) were in similar proportions among all west Atlantic samples, including the Greenland Sisimiut cod. Population pair-wise *F*_ST _values based on all six SNP loci demonstrated a clear genetic separation between eastern and western cod samples, with intermediate Northeast Arctic (Båtsfjord) and Greenland cod (Figure 7; Additional file 4: Table S2). Heterozygosity showed an increasing trend from almost zero in the Baltic to close to 50% in western Atlantic samples (Table 1, Additional file 3: Table S1).

**Table 1:** Atlantic cod sampling locations and heterozygosities.

Locality	Lat	Long	Sampling year	Sample size	***H***_**o**_	***H***_**e**_
Baltic Öland	56.04	16.41	2004	29	0.006	0.006
Baltic Bornholm	55.50	16.00	2004	30	0.039	0.039
Kattegat	56.90	12.15	2004	29	0.058	0.056
North Sea	55.57	05.85	2002	29	0.126	0.119
Norwegian coast, Molde	62.80	06.44	2003	12	0.083	0.083
Norwegian coast, Malangen	69.71	17.33	2003	18	0.222	0.203
Faeroe Bank	61.10	-08.30	2008	50	0.154	0.143
Faeroe Plateau	61.96	-06.02	2008	49	0.109	0.104
Barents Sea, Båtsfjord	70.65	29.81	2003	10	0.220	0.304
Greenland Nuuk	64.73	-50.45	2003	25	0.419	0.344
Greenland Sisimiut	66.84	-52.89	2003	25	0.573	0.469
Labrador	52.06	-53.39	2004	19	0.534	0.466
Nova Scotia	45.74	-58.41	2003	25	0.456	0.459
Georges bank	42.15	-67.01	2003	25	0.546	0.478

*H*_o _observed heterozygosity across six *Tf1 *SNP loci, *H*_e _expected heterozygosity.

## Discussion

The identification of four transferrin genes in the Atlantic cod genome adds novel information to our knowledge about the evolution of the transferrin gene family by documenting 1) the expression of tandem duplicated transferrin genes, 2) presence of both bilobal and monolobal transferrin forms in the fish genome, 3) highly conserved synteny between fish monolobal and tetrapod bilobal transferrin loci, and 4) evidence of positive selection acting on a tandem duplicated transferrin gene in trans-Atlantic cod populations. The close linkage of the *Tf1 *and *Tf2 *genes in the cod genome confirms the suggested tandem duplication of transferrin genes in salmonids and channel catfish based on Southern blotting, pedigree and RFLP analyses [45-48]. The higher sequence identity between the Tf genes in salmonids (96%) than in cod (84%) indicates separate events of tandem gene duplication of this iron-binding protein, and are supported by the separate TF1/Tf2 bifurcations in salmonids, cod and flounder in the phylogenetic tree.

The evolution of bilobal transferrins from an ancestral monolobal form is thought to have occurred in the last common ancestor of vertebrates and arthropods at least 600 MYA [28,49-51]. However, Williams et al. [52] proposed that the gene duplication event occurred in the prochordates about 500 MYA and correlated with the evolution of a filtration kidney in higher vertebrates that would cause the excretion of monolobal transferrins of about 40 kDa. Tinoco et al. [24] argued that the major advantage for the evolution of bilobal transferrins is the stronger iron affinity because of cooperativity, while monolobal transferrins may have evolved as more general metal ion transporters such as the proposed role of ascidian nicaTf in vanadium trafficking using carbonate as synergistic anion [53]. Contrasting with nicaTf, the iron-binding residues are not conserved in the fish OMP, which seems to be involved in the otolith formation by providing calcium carbonate [25]. The otoliths grow by the continuous deposition of calcium carbonate, and knockdown of zebrafish *Omp *caused a reduction in otolith size [27]. Thus, this monolobal fish transferrin form has apparently acquired a novel function within the otoliths without being excreted by the kidney.

The evolution of modern transferrins might be elucidated by searching for conserved synteny between monolobal and bilobal transferrin genes. Similar to the linkage of the different transferrin genes in mammals and chicken [54], the *Ciona nicaTf *and *Tf*-like genes are both located on chromosome 7q [43], but we were not able to find any syntenic segments between these linkage groups. In contrast, the fish *Tf*, *MTf *and *Omp *genes are positioned on three linkage groups, and the *Omp *flanking genes *Senp2*, *Tra2b *and *D2hgdh *are closely linked to chicken *Tf *on chromosome 9, while *Senp2*, *Tra2b *and *Slitrk3 *are flanked by human *MTF *and *Tf *on chromosome 3 (Figure 2). The conserved synteny between fish monolobal and tetrapod bilobal transferrin loci infers close evolutionary relationship, and we propose that an *Omp*-like gene became duplicated and gave rise to a bilobal form in the common ancestor of the fish and tetrapod lineages. The presence of a single *Omp *gene and the chromosomal separation of the different transferrin genes in the extant fish genome could be explained by two whole genome duplication events, which are believed to have occurred in the ancestral vertebrate [55]. Our speculative model implies that the melanotransferrin and the other transferrins did not evolve from a common bilobal protein, but originated from at least two distinct single gene duplication and fusion events. This is supported by the much higher similarity between the N- and C-lobes of melanotransferrin compared to serum transferrin, although the difference might also be explained by their different functions [56].

The success of competition for the transferrin-bound iron by pathogens depends on the shape and surface charge of the transferrin molecule [57]. Selection for new replacement alleles has been proposed to play a large role in the evolution of transferrin within salmonids, carp and notothenioids [35,58-60]. We show here that positive selection on the *Tf1 *gene in Atlantic cod is significant as evidenced from the high *d*_*N*_/*d*_*S *_ratio between western and eastern Atlantic cod sequences, and suggest that the replacement of surface residues might affect the binding of pathogen transferrin receptors. Another way to test for positive selection is to compare patterns of genetic divergence among populations for the transferrin SNPs with the population structure in neutral genetic markers. Two microsatellite datasets [61,62] and one dataset based on 88 putatively neutral SNPs [13] cover most of the transect from the Baltic along the Norwegian coast across to Greenland, Canada and USA. These neutral markers show several times less divergence compared to the transferrin SNPs (Figure 7). Although one should be cautious when comparing genetic markers with different mutational properties such as microsatellites and SNPs, this large difference would suggest positive selection on the transferrin gene, rather than divergence due to geographic isolation. Atlantic cod has been shown in previous studies to be subject to positive selection on several genetic loci in relation to different environmental factors on both local and global scales. Most notable are the observed clines in PanI and hemoglobin in relation to water temperature [9,11,63,64]. Baltic cod is highly divergent in the hemoglobin [65] and heat shock protein *HSP90 *genes [13] compared to other populations, and show specific adaptations such as egg buoyancy and sperm motility to the low salinity environment [66]. Intriguingly, additional biological functions of transferrin were suggested by the relation of the carp transferrin polymorphism to sperm motility characteristics [67], and the transferrin polymorphism in tilapia was proposed to be associated with saltwater tolerance [68]. However, transferrin does not seem to be under a particular selection pressure in the Baltic cod population, since SNP allele frequencies in Baltic samples were similar to those collected in the Kattegat, Skagerrak and North Sea.

In a recent large scale population genomic study of 1641 SNP loci, Bradbury et al. [14] demonstrated temperature-associated clines in SNP allele frequencies on both sides of the Atlantic, suggesting selection on multiple independent genes in response to ocean temperature. This large-scale study also showed a clear pattern of reduced heterozygosity in eastern cod populations [14]. Whereas a similar cline in heterozygosity across the Atlantic was shown in the present tranferrin study, Nielsen et al. [13] did not find such a pattern for 88 neutral SNP loci. The contrasting patterns of heterozygosity may be due to ascertainment bias, since the SNP loci showing reduced heterozygosity in the eastern Atlantic were developed from Canadian cod [14], whereas the SNPs in Nielsen et al. [13] were developed from Norwegian cod in the east Atlantic. In the present study, the transferrin SNPs were identified by comparing cDNA sequences from Canadian and Norwegian cod specimen, thus excluding ascertainment bias due to different population histories. Instead the difference in heterozygosity between eastern and western Atlantic cod is most likely due to selection. The issue whether selection has acted on the NE or NW cod populations or both might be addressed by comparing the different gadoid Tf sequences. The phylogenetic analysis showed that the Tf1-NE variant had higher similarity than the NW variant to the closest outgroups haddock Tf and cod Tf2. Comparison of the 16 aa changing sites in the cod Tf1 shows that the NE variant is identical to haddock Tf in 8 sites, while only 3 sites in the NW variant are identical to haddock Tf (Table 2). In addition, the NE variant of Tf1 is identical to Tf2 at 12 sites, whereas the NW variant and Tf2 are identical at only one site of the 16 substitutions. Thus, the most parsimonious explanation is that the NE variant is ancestral to the NW variant of cod Tf1, and that cod in the NW populations has undergone adaptive evolution. If environmental conditions vary temporally, multiple alleles may be selected for, increasing heterozygosity, as has been suggested for immune resistance genes [69,70]. We have not investigated polymorphism in cod Tf2 in the different populations, which might further elucidate the origin and divergence of this iron-binding protein.

**Table 2:** Comparison of the 16 substituted amino acids in the two cod Tf1 variants with cod Tf2 and haddock Tf

SNP					tf-6	tf-8			tf-10	tf-11	tf-13						tf-22
**AA site**	**52**	**160**	**161**	**264**	**270**	**280**	**285**	**286**	**337**	**369**	**407**	**475**	**653**	**668**	**680**	**686**	**688**

Cod TF1-NE	**R**	E	A	**D**	G	R	**N**	L	T	Y	G	**T**	Q	V	D	T	F
Cod TF1-NW	K	S	-	V	*E*	T	*S*	F	P	S	Q	H	L	*I*	E	I	S
Cod TF2	**R**	E	A	**D**	R	R	**N**	L	-	N	G	**T**	Q	*I*	D	T	F
Haddock	-	E	A	K	*E*	R	*S*	L	S	Q	G	I	Q	*I*	D	T	F

Ala161 is deleted in cod Tf1-NW.

Could historical factors have shaped the cod *Tf1 *SNP allele frequencies? Paleoecological modelling, as well as nuclear and mitochondrial genetic markers, suggests that cod populations have survived as least for 100 000 years on both sides of the Atlantic [36,37]. Cod populations were more fragmented in the western Atlantic during the last glacial maximum 20 KYA [36], and due to drift and bottlenecks different alleles may have increased in frequency in more or less isolated NW populations. However, the higher substitution rate in nonsynonymous sites, and the fact that multiple alleles are present also in the NE populations, though in low frequencies, argues against the idea that historical expansion in the NW populations alone could explain the observed genetic pattern. Other genetic data suggest that the Greenland populations of Atlantic cod post-dates the last glacial period [36], which possibly could help explain the observed intermediate allele frequencies in Greenland samples.

## Conclusion

The fish transferrin genes provide novel evidence for the evolution of modern transferrins from monolobal transferrins by documenting highly conserved synteny between fish and tetrapods. We propose that the evolution of the iron-binding and iron-independent functions of the different transferrin family members probably involved more than a single event of gene duplication and fusion of monolobal transferrins. Although the specific forces driving evolution of the cod Tf1 are uncertain, the multiple surface residue changes suggest an evolutionary competition for transferrin-bound iron between the host and invading pathogens. We did not find any difference in transferrin allele frequency between the two Faroe populations as previously suggested [39], and also recently based on microsatellites [61]. Neither was the distinctness of Baltic cod previously demonstrated for both neutral markers and coding genes, eg. hemoglobin, supported by the transferrin polymorphisms. Our results underline that functional genetic differences should not be overlooked in fisheries management, but also that markers under selection may give a completely different view of stock structure compared to neutral markers.

## Methods

### Identification of cod transferrin genes and alleles

The genomic sequences of Atlantic cod *Tf1*, *Tf2*, *MTf *and *Omp *were found by BLAST search of the reference genome representing the Northeast Arctic population (http://www.codgenome.no), and the cDNAs were identified by screening the GAFFA database of the Norwegian coastal cod population. The designated NE variant of cod *Tf1 *differed at multiple positions from a published cod *Tf *sequence [44]. This was verified by PCR using templates of liver cDNA and genomic DNA from three Faroe cod specimen, including one heterozygous fish possessing both variants. Primer sequences are available upon request. The liver samples were stored in RNA later (Ambion) before extraction of genomic DNA (Qiagen) and total RNA using Trizol Reagent (Invitrogen Life Technologies). Liver cDNA was synthesized from 1 μg total RNA using the SMART RACE cDNA amplification kit (Clontech Laboratories Inc.) after DNAse treatment (TURBO DNA-free kit, Ambion). The PCR reactions were cycled in a standard thermocycler using the Advantage 2 PCR Enzyme system (Clontech) at standard conditions recommended by the manufacturer. The PCR products were sequenced in both directions with Big Dye sequencing kit (v.3.1) on 3730 ABI DNA Analyser (Applied Biosystems).

### Gene expression analyses

#### RT-PCR

Tissue expression of *Tf1 *and *Tf2 *was semi-quantified by reverse transcription (RT)-PCR using liver and brain from two adult Atlantic cod from the Northeast Arctic population. PCR was run for 35 or 25 cycles with gene specific primers (Additional file 5: Table S3) on liver and brain cDNA templates synthesized as described above.

#### Whole mount in situ hybridization (WISH)

WISH analysis of *Omp *expression in cod larvae was carried out as described [71]. A 906-bp region of cod *Omp *was PCR amplified using gene specific primers (Additional file 5: Table S3), and sense- and antisense probes, respectively, were synthesized from Sp6- and T7-tailed PCR product and labelled with digoxigenin (Roche, Basel, Switzerland). Twenty newly hatched larvae were fixed for WISH analysis.

### Generation of 3D protein structures

All computational experiments were conducted on a Hewlett-Packard xw8600 workstation running Red Hat Enterprise Linux 5. Sequence analysis was performed using BLAST [72] through the Protein Data Bank (BLOSUM62 matrix). Human serum transferrin (Protein Data Bank ID 2HAU) [73] was selected as the most appropriate template to generate comparative models and showed sequence identities with cod Tf1-NE, Tf1-NW, Tf2, MTf and OMP of 45%, 45%, 44%, 40% and 35%, respectively. The template structure was then aligned against the cod sequences using ClustalW program [74]. Each sequence alignment was then checked to ensure that (i) all the secondary structural elements had a minimum number of insertions or deletions within them, and (ii) Cys residues forming consensus disulfide bridges were conserved [75]. Once an acceptable alignment had been produced, an ensemble of 50 models of the five different cod proteins were built using MODELLER v. 9.7 [76] as implemented in Discovery Studio (Accelrys Inc., San Diego, CA, USA) and ranked using the MODELLER objective function, which is highly efficient in ranking different models calculated from the same alignment. It should be noted that the disulfide bridges between conserved Cys were not included as a restraint in the modeling process, but that the relative position of two Cys side chains in the resulting (unrestrained) model led us to suggest the existence of a disulfide. The stereochemical quality of the structures was assessed by PROCHECK [77] supplemented by the profile programs VERIFY3D [78] and ProSA-web [79]. In order to assess the reliability of each model, the corresponding energy graphs were compared with the template 2HAU structure.

### Phylogenetic analyses

Database searches were used to identify in total 40 amino acid sequences from teleost species, *Ciona intestinalis *and man (Additional file 6: Table S4). The C-terminal domain of Tf and MTf sequences was aligned with OMP sequences using ClustalX [80] and by manual editing. The final alignment consisted of 40 taxa and 330 characters. The best evolution model based on the sequence alignment was determined using ProtTest [81]. The sequences were used to infer the phylogeny in a Bayesian framework applying the program MrBayes v3.1.2 [82]. The Bayesian inferences were done as follows: two independent runs, each with three cold and one heated MCMC (Markov Chain Monte Carlo) chains were started from a random starting tree. The two runs lasted for 5,000,000 generations. The covarion (COV) model was used together with the WAG+G+I to accommodate for different substitution rates across sites (G + proportion of invariable sites (I)) and across sequences (COV). The maximum likelihood (ML) tree was estimated using the program RAxML v.6 [83]. The topology with the highest likelihood score out of 100 heuristic searches, each from a random starting tree, was selected, and bootstrapping was done with 100 pseudoreplicates and one heuristic search per replicate. In the ML analyses, the WAG model with a gamma-distributed rate of variation across sites (G) was employed. All phylogenetic analyses were done on the freely available Bioportal at University of Oslo http://www.bioportal.uio.no. The Nei-Gojobori method [84] implemented in DNAsp 5.0 was used to calculate rates of synonymous and non-synonymous substitutions in the identified Norwegian *Tf1 *cDNA sequence compared to the Canadian sequence [44]. We then used the *z*-test in MEGA (4.0) to test for positive selection.

### Population sampling and SNP genotyping

In total 375 adult individuals were collected during 2002-2008 from 14 localities covering the geographical region of the North Atlantic inhabited by Atlantic cod (Table 1, Additional file 2; Figure S2). Genomic DNA was extracted from fin clips, muscle tissue or gill arches, and six SNPs loci (Additional File 1: Figure S1) spanning the *Tf1 *gene were genotyped for all individuals using the MassARRAY system from Sequenom (San Diego, USA). PCR primers and extension primers were designed using the software SpectroDESIGNER v3.0 (Sequenom) (Additional file 5: Table S3). Genomic DNA was PCR amplified as described [85], and the SNP genotyping was performed according to the iPLEX protocol from Sequenom available at http://www.sequenom.com/iplex. For allele separations the Sequenom MassARRAY Analyzer (Autoflex mass spectrometer) was used. Genotypes were assigned in real time [86] using the MassARRAY SpectroTYPER RT v3.4 software (Sequenom) based on the mass peaks present. All results were manually inspected using the MassARRAY Typer Analyzer v3.3 software (Sequenom).

### Statistical analyses of population genetic data

SNP heterozygosities and population differentiation (*F*_ST_) were calculated using Genepop 4.0.10 [87]. Statistical significance of population differentiation was assessed using Fishers exact test implemented in Genepop.

## List of abbreviations

Tf: Transferrin; OMP: otolith matrix protein; Mtf: melanotransferrin; RMSD: root mean square deviation; WISH: whole mount in situ hybridization; RT-PCR: reverse transcription polymerase chain reaction; UTR: untranslated region; ORF: open reading frame.

## Authors' contributions

ØA and CA conceived and designed the study, and wrote the manuscript. MCDR and DP performed the computer modeling study and the generation of 3D structures. ATK carried out the phylogenetic analyses. PEP was responsible for the Faroe cod samples. CA performed the statistical analyses. All authors critically read the manuscript drafts and approved the final version of the manuscript.

## Supplementary Material

Additional file 1 **Figure S1 Alignment of the NE and NW variants of Atlantic cod *Tf1 *cDNAs**. The Atlantic cod Tf1 cDNA derived from two Northeast Atlantic populations was compared with that of a Northwest Atlantic population [44]. The 22 SNPs identified are numbered, and the six analysed SNPs are shown in bold. Click here for file

Additional file 2 **Figure S2 Map of the Atlantic cod populations examined**. Six out of 22 SNPs identified in cod Tf1 were analysed in 14 populations across the North-Atlantic. Click here for file

Additional file 3 **Table S1 Genotype frequencies for six Atlantic cod Tf1 SNP loci in 14 samples across the North Atlantic**. Sample sizes of cod collected at each location in the first row. Click here for file

Additional file 4 **Table S2 Genetic differentiation among pairs of Atlantic cod samples**. Below diagonal are pairwise *F*_ST_-values, and above diagonal statistical significance values *P < 0.05; **P < 0.01; ***P < 0.001. Click here for file

Additional file 5 **Table S3 PCR primers for SNP analysis, RT-PCR and WISH**. The sequences are shown in 5'-3' direction. Click here for file

Additional file 6 **Table S4 Accesion numbers of the proteins included in the phylogenetic analysis**. Several distinct transferrin genes have been described in some teleost species. Click here for file
