# Supplementary material for: Polymorphism, selection and tandem duplication of transferrin genes in Atlantic cod (Gadus morhua) - Conserved synteny between fish monolobal and tetrapod bilobal transferrin loci
Source: BMC Genet. 2011 May 25;12:51. doi: 10.1186/1471-2156-12-51 (PMC3125230; doi:10.1186/1471-2156-12-51)
Supplement: Additional file 1 — Figure S1 Alignment of the NE and NW variants of Atlantic cod Tf1 cDNAs. The Atlantic cod Tf1 cDNA derived from two Northeast Atlantic populations was compared with that of a Northwest Atlantic population [44]. The 22 SNPs identified are numbered, and the six analysed SNPs are shown in bold. [file 1471-2156-12-51-S1.DOC]

NE ATGAAGCATCTCCTCCTGTCGGTGCTGTTTTGCTGTCTGGCAACTGCCTTCAGCGTGCCTGTAGAAGTGGTGAGGTGGTGCGTGACCTCACCTTTCGAGAAGCGGAAATGTGATGCCCTG 120

||||||||||||||||||||||||||||||||||||||||||||||||||||||||||||||||||||||||||||||||||||||||||||||||||||||||||||||||||||||||

NW1 ATGAAGCATCTCCTCCTGTCGGTGCTGTTTTGCTGTCTGGCAACTGCCTTCAGCGTGCCTGTAGAAGTGGTGAGGTGGTGCGTGACCTCACCTTTCGAGAAGCGGAAATGTGATGCCCTG 120

1

NE AAGCTTAGGCAACCGGTGTTCACATGCGTATTAAGAGCAGACGCCACTGAGTGCATTTTGGCAATCAAGGCCGGTGAAGCAGATGCCATCACATTGGACGGAGGGGAGATCTACACAGCA 240

|||||||||||||||||||||||||||||||||| |||||||||||||||||||||||||||||||||||||||||||||||||||||||||||||||||||||||||||||||||||||

NW AAGCTTAGGCAACCGGTGTTCACATGCGTATTAAAAGCAGACGCCACTGAGTGCATTTTGGCAATCAAGGCCGGTGAAGCAGATGCCATCACATTGGACGGAGGGGAGATCTACACAGCA 240

NE GGACAGCACCCCTATGATCTGCAGCCCATTATTTCTGAGAAATATGGCTCAGGGTCCTCCTGCTACTACGCTGTGGCTGTCGTGAAGAAAGACACTGGATTCTCCTTCAAACAGCTGAGA 360

||||||||||||||||||||||||||||||||||||||||||||||||||||||||||||||||||||||||||||||||||||||||||||||||||||||||||||||||||||||||

NW GGACAGCACCCCTATGATCTGCAGCCCATTATTTCTGAGAAATATGGCTCAGGGTCCTCCTGCTACTACGCTGTGGCTGTCGTGAAGAAAGACACTGGATTCTCCTTCAAACAGCTGAGA 360

NE GGGAAGAAATCGTGCCACACCGGTATTGGGAAGACCGCGGGCTGGAACATTCCCATCGGAACTCTTCTGACGACGGGTCAACTCGTTTGGAGCGGGCAGGAGGACTTGCCTGTGGAAGAG 480

|||||||||||||||||||||||||||||||||||||||||||||||||||||||||||||||||||||||||||||||||||||||||||||||||||||||||||||||||||||

NW GGGAAGAAATCGTGCCACACCGGTATTGGGAAGACCGCGGGCTGGAACATTCCCATCGGAACTCTTCTGACGACGGGTCAACTCGTTTGGAGCGGGCAGGAGGACTTGCCTGTGGAA--- 478

234

NE GCGGTGAGCACCTTCTTCTCGAAGAGCTGCGTTCCCGGAGCAGGGGGTCTGGTTGGCGGTAAACTGTGCACATTGTGCCCGAGTGACTGCAGCAAGTCGGCCACTAACCCCTACTTTGGA 600

|||||||||||||||||||||||||||||||||||||||||||||||||||||||||||||||||||||||||||||||||||||||||||||||||||||||||||||||||||||

NW AGCGTGAGCACCTTCTTCTCGAAGAGCTGCGTTCCCGGAGCAGGGGGTCTGGTTGGCGGTAAACTGTGCACATTGTGCCCGAGTGACTGCAGCAAGTCGGCCACTAACCCCTACTTTGGA 597

NE TACGCAGGAGCATTCAAGTGTCTGAAAGACGATGCCGGAGACGTTGCCTTTATCAACCACCTCACAGTTCCAGCGTCAGAGAAAGCCAACTACGAGCTGCTGTGCCTGGATGGTACGAGA 720

||||||||||||||||||||||||||||||||||||||||||||||||||||||||||||||||||||||||||||||||||||||||||||||||||||||||||||||||||||||||

NW TACGCAGGAGCATTCAAGTGTCTGAAAGACGATGCCGGAGACGTTGCCTTTATCAACCACCTCACAGTTCCAGCGTCAGAGAAAGCCAACTACGAGCTGCTGTGCCTGGATGGTACGAGA 717

5 **6** 7

NE GCTCCCATCGACAGCTACAAGACCTGTAACCTGGCCAGGGTTCCCGCCCACGCTGTGGTTAGCCGCGATGACCCCGAACTAGCCGGGCGTATCTTCACCGCCCTCACCACCGTCAGGGGC 840

||||||||||||||||||||||||||||||||||||||||||||||||||||||||||||||||||| ||||||||||||||||| ||||||||||||||||||||||||||||| ||||

NW GCTCCCATCGACAGCTACAAGACCTGTAACCTGGCCAGGGTTCCCGCCCACGCTGTGGTTAGCCGCGTTGACCCCGAACTAGCCGAGCGTATCTTCACCGCCCTCACCACCGTCACGGGC 837

**8** 9

NE TTTAACCTCTTCTCCTCTGCTGGGTTTGGTGCTGCGAACTTGATGTTCAAAGACACAACACAGAGTCTTGTCAGGCTGCCCGACGGCAGCAACTCCTTCCTCTACCTGGGCGCTAAATAC 960

|||| | |||||||||||||||||||||||||||||||||||||||||||||||||||||||||||||||||||||||||||||||||||||||||||||||||||||||||||||||||

NW TTTAGCTTCTTCTCCTCTGCTGGGTTTGGTGCTGCGAACTTGATGTTCAAAGACACAACACAGAGTCTTGTCAGGCTGCCCGACGGCAGCAACTCCTTCCTCTACCTGGGCGCTAAATAC 957

**10**

NE ATGGCGTCCATCCAATCTCTGAAGAAAGAATCGGATCAAACAATAACGCCTGCGATCAAATGGTGCGCTGTGGGTCACGCTGAGAAGAAAAAGTGTGACTCATGGAGTTCATTCAGCGTC 1080

||||||||||||||||||||||||||||||||||||||| ||||||||||||||||||||||||||||||||||||||||||||||||||||||||||||||||||||||||||||||||

NW ATGGCGTCCATCCAATCTCTGAAGAAAGAATCGGATCAACCAATAACGCCTGCGATCAAATGGTGCGCTGTGGGTCACGCTGAGAAGAAAAAGTGTGACTCATGGAGTTCATTCAGCGTC 1077

**11** 12

NE TCCGATGGAGTCAAATACGTTGCATGTCAGATTAGCCTAACAGTGGAGGGCTGCTTTCAGAGGATTATGCGTCAAGAGGCTGATGCCATGTCTGTAGATGGAGGACAGGTGTACACCGC 1199

||||||||||||||| ||||||||||||||||||||||||||||||||||||||||||||||||||||||||||||||||||||||||||||||||||||||||||||||||||||||

NW TCCGATGGAGTCAAAAGCGTTGCATGTCAGATTAGCCTAACAGTGGAGGGCTGCTTTCAGAGGATTATGCGTCAAGAGGCTGATGCCATGTCTGTAGATGGAGGACAGGTGTACACCGC 1196

**13** 14

NE TGGGAAGTGTGGACTGATCCCAGCCATGGTCGAGCAATACAATCAATCACTTTGCAGTTCTGCCGGAACTCCTCAGGCCACATACTTCGCCGTTGCCGTGGTGAAGAAGGGCTCCGGGGT 1319

|||||||||| ||||||||||||||||||||||||||||||||||||||||||||||||||||||||||||||||||||||||||||||||||||||||||||||||||||||||||||

NW TGGGAAGTGTCAACTGATCCCAGCCATGGTCGAGCAATACAATCAATCACTTTGCAGTTCTGCCGGAACTCCTCAGGCCACATACTTCGCCGTTGCCGTGGTGAAGAAGGGCTCCGGGGT 1316

15 16

NE GACCTGGGACAACCTGAGGGGTAAGAGGTCCTGCCACACGGGCTTGGGCAGAACCGCCGGCTGGAACATCCCGATGGGCCTGGTCCACTCCATCACCGGAAGCTGTGACTTCGGTGGTT 1438

|||||||||||||||||||||||||||||||||||||||||||||||||||||||||||||||||||||||||||||||||||||||||||||| |||||||||||||||||||||||

NW GACCTGGGACAACCTGAGGGGTAAGAGGTCCTGCCACACGGGCTTGGGCAGAACCGCCGGCTGGAACATCCCGATGGGCCTGGTCCACTCCATCCACGGAAGCTGTGACTTCGGTGGTT 1435

NE TCTTCCCCAGTGGCTGTGCCCCGGGGTCTGAACCCTCATCCACATTCTGTCGACAGTGTGCCGGCAGCGGATCTGGAGTGGAAGATGGTTCCAAGTGCAGCGCCAGCTCTGTAGAGAAGT 1558

||||||||||||||||||||||||||||||||||||||||||||||||||||||||||||||||||||||||||||||||||||||||||||||||||||||||||||||||||||||||

NW TCTTCCCCAGTGGCTGTGCCCCGGGGTCTGAACCCTCATCCACATTCTGTCGACAGTGTGCCGGCAGCGGATCTGGAGTGGAAGATGGTTCCAAGTGCAGCGCCAGCTCTGTAGAGAAGT 1555

NE ACTACGGCTACGCTGGAGCGTTCAGATGTCTTGTTGACGGTGCCGGGGATGTTGCCTTTATTAAACACACTATTGTGGCAGATAACAGCGATGGACAAGGTCCAGCCTGGGCTACAGCAT 1678

||||||||||||||||||||||||||||||||||||||||||||||||||||||||||||||||||||||||||||||||||||||||||||||||||||||||||||||||||||||||

NW ACTACGGCTACGCTGGAGCGTTCAGATGTCTTGTTGACGGTGCCGGGGATGTTGCCTTTATTAAACACACTATTGTGGCAGATAACAGCGATGGACAAGGTCCAGCCTGGGCTACAGCAT 1675

NE TGAAATCTTCAGACTATCAACTAATTTGCCCTGGGGGTGTTGGTAGGGCAGAGATAAGCGACTTTGCCTCGTGCAACCTGGCCGCAGTTCCTTCCCATGCTGTTGTGACGCGCCAAGACA 1798

||||||||||||||||||||||||||||||||||||||||||||||||||||||||||||||||||||||||||||||||||||||||||||||||||||||||||||||||||||||||

NW TGAAATCTTCAGACTATCAACTAATTTGCCCTGGGGGTGTTGGTAGGGCAGAGATAAGCGACTTTGCCTCGTGCAACCTGGCCGCAGTTCCTTCCCATGCTGTTGTGACGCGCCAAGACA 1795

NE TTCGCGACGATGTGGTGAAGATGCTCCTCGACCAACAGCGCAAGTTCGGTATAGATGGTAGCGATCCTTTATTCAGGATTTACGAATCAAAAGATGGAAATAACCTCCTCTTTAAAGACT 1918

||||||||||||||||||||||||||||||||||||||||||||||||||||||||||||||||||||||||||||||||||||||||||||||||||||||||||||||||||||||||

NW TTCGCGACGATGTGGTGAAGATGCTCCTCGACCAACAGCGCAAGTTCGGTATAGATGGTAGCGATCCTTTATTCAGGATTTACGAATCAAAAGATGGAAATAACCTCCTCTTTAAAGACT 1915

17 18 19 20

NE CCACTAAGTGTCTCAAGGAGATACCAAGTCAAACTACAGCCGATGCTTTCCTGGGAACTGGCTACGTGAACGCCGTCATGTCCCTCCGGCAGTGCCCTGAGACTGCTTCTGATCTGGAGA 2038

||||||||||||||||||||||||||||| ||||||||||||||||||||||||||||||||||||||||||| ||||||||||||||||||||||||||||||||||||| |||||||

NW CCACTAAGTGTCTCAAGGAGATACCAAGTTTAACTACAGCCGATGCTTTCCTGGGAACTGGCTACGTGAACGCCATCATGTCCCTCCGGCAGTGCCCTGAGACTGCTTCTGAACTGGAGA 2035

21 **22**

NE AAACCTGCACCTCCTTTTCCTGCAGTACTGCAGAATGA 2076

||||||||| ||||| ||||||||||||||||||||||

NW AAACCTGCATCTCCTCTTCCTGCAGTACTGCAGAATGA 2073
