# Supplementary figures and images for: Polymorphism, selection and tandem duplication of transferrin genes in Atlantic cod (Gadus morhua) - Conserved synteny between fish monolobal and tetrapod bilobal transferrin loci
Source: BMC Genet. 2011 May 25;12:51. doi: 10.1186/1471-2156-12-51 (PMC3125230; doi:10.1186/1471-2156-12-51)

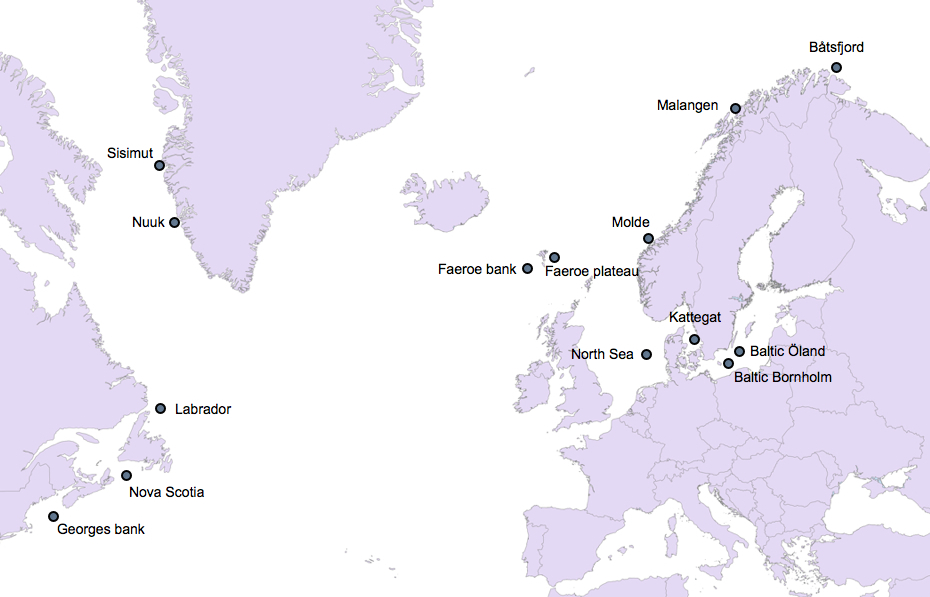

Supplement: Additional file 2 — Figure S2 Map of the Atlantic cod populations examined. Six out of 22 SNPs identified in cod Tf1 were analysed in 14 populations across the North-Atlantic. [file 1471-2156-12-51-S2.DOC]
