# Supplementary material for: Polymorphism, selection and tandem duplication of transferrin genes in Atlantic cod (Gadus morhua) - Conserved synteny between fish monolobal and tetrapod bilobal transferrin loci
Source: BMC Genet. 2011 May 25;12:51. doi: 10.1186/1471-2156-12-51 (PMC3125230; doi:10.1186/1471-2156-12-51)
Supplement: Additional file 3 — Table S1 Genotype frequencies for six Atlantic cod Tf1 SNP loci in 14 samples across the North Atlantic. Sample sizes of cod collected at each location in the first row. [file 1471-2156-12-51-S3.DOC]

|  | **Baltic** | **Baltic** | **Kattegat** | **North** | **Molde** | **Malangen** | **Faeroe** | **Faeroe** | **Båtsfjord** | **Greenland** | **Greenland** | **Labrador** | **Nova** | **Georges** |
| --- | --- | --- | --- | --- | --- | --- | --- | --- | --- | --- | --- | --- | --- | --- |
|  | **Öland** | **Bornholm** |  | **Sea** |  |  | **bank** | **plateau** |  | **Nuuk** | **Sisimiut** |  | **Scotia** | **bank** |
|  |  |  |  |  |  |  |  |  |  |  |  |  |  |  |
| (n) | 29 | 30 | 29 | 29 | 12 | 18 | 50 | 49 | 10 | 25 | 25 | 19 | 25 | 25 |
| **tf-6** |  |  |  |  |  |  |  |  |  |  |  |  |  |  |
| GG | 29 | 29 | 28 | 26 | 11 | 14 | 43 | 44 | 7 | 13 | 8 | 3 | 5 | 5 |
| GA | 0 | 1 | 1 | 3 | 1 | 4 | 7 | 5 | 2 | 11 | 17 | 8 | 13 | 16 |
| AA | 0 | 0 | 0 | 0 | 0 | 0 | 0 | 0 | 0 | 0 | 0 | 0 | 0 | 0 |
| **tf-8** |  |  |  |  |  |  |  |  |  |  |  |  |  |  |
| AA | 29 | 29 | 28 | 26 | 11 | 14 | 43 | 44 | 7 | 14 | 8 | 3 | 5 | 5 |
| AG | 0 | 1 | 1 | 3 | 1 | 4 | 7 | 5 | 2 | 11 | 14 | 7 | 7 | 10 |
| GG | 0 | 0 | 0 | 0 | 0 | 0 | 0 | 0 | 1 | 0 | 3 | 9 | 13 | 10 |
| **tf-10** |  |  |  |  |  |  |  |  |  |  |  |  |  |  |
| AA | 29 | 29 | 28 | 26 | 11 | 14 | 43 | 44 | 7 | 12 | 8 | 2 | 4 | 4 |
| AC | 0 | 1 | 1 | 3 | 1 | 4 | 7 | 5 | 2 | 12 | 14 | 8 | 8 | 11 |
| CC | 0 | 0 | 0 | 0 | 0 | 0 | 0 | 0 | 1 | 0 | 2 | 9 | 12 | 10 |
| **tf-11** |  |  |  |  |  |  |  |  |  |  |  |  |  |  |
| TT | 28 | 28 | 24 | 22 | 11 | 14 | 38 | 41 | 7 | 14 | 12 | 2 | 3 | 3 |
| TA | 1 | 2 | 5 | 7 | 1 | 4 | 11 | 8 | 2 | 10 | 9 | 6 | 7 | 10 |
| AA | 0 | 0 | 0 | 0 | 0 | 0 | 0 | 0 | 1 | 1 | 3 | 11 | 15 | 10 |
| **tf-13** |  |  |  |  |  |  |  |  |  |  |  |  |  |  |
| GG | 29 | 29 | 28 | 26 | 11 | 14 | 43 | 44 | 7 | 16 | 8 | 4 | 7 | 8 |
| GC | 0 | 1 | 1 | 3 | 1 | 4 | 7 | 4 | 3 | 7 | 17 | 15 | 18 | 17 |
| CC | 0 | 0 | 0 | 0 | 0 | 0 | 0 | 0 | 0 | 0 | 0 | 0 | 0 | 0 |
| **tf-22** |  |  |  |  |  |  |  |  |  |  |  |  |  |  |
| TT | 29 | 29 | 28 | 26 | 11 | 14 | 43 | 44 | 7 | 13 | 8 | 4 | 6 | 8 |
| TC | 0 | 1 | 1 | 3 | 1 | 4 | 7 | 5 | 2 | 9 | 14 | 11 | 10 | 14 |
| CC | 0 | 0 | 0 | 0 | 0 | 0 | 0 | 0 | 1 | 0 | 3 | 4 | 9 | 3 |
