# Supplementary material for: Polymorphism, selection and tandem duplication of transferrin genes in Atlantic cod (Gadus morhua) - Conserved synteny between fish monolobal and tetrapod bilobal transferrin loci
Source: BMC Genet. 2011 May 25;12:51. doi: 10.1186/1471-2156-12-51 (PMC3125230; doi:10.1186/1471-2156-12-51)
Supplement: Additional file 5 — Table S3 PCR primers for SNP analysis, RT-PCR and WISH. The sequences are shown in 5'-3' direction. [file 1471-2156-12-51-S5.DOC]

| SNP_ID | Forward primer | Reverse primer | Extension primer |
| --- | --- | --- | --- |
| tf-6 | ACGTTGGATGGTGTGTGCATGCGTATTACC | ACGTTGGATGTCCCGCCCACGCTGTGGTTA | TGACCCCGAACTAGCCG |
| tf-8 | ACGTTGGATGTTACTTGTTCACTCTGCAGG | ACGTTGGATGGAACATCAAGTTCGCAGCAC | CAGCAGAGGAGAAGAGG |
| tf-10 | ACGTTGGATGTTTTCTTCTCAGCGTGACCC | ACGTTGGATGACCTTACCTCGACTCCATAG | CTCCATAGAATCGGATCAA |
| tf-11 | ACGTTGGATGTAATCCTCTGAAAGCAGCCC | ACGTTGGATGTGGAGTTCATTCAGCGTCTC | CGTCTCCGATGGAGTCAAA |
| tf-13 | ACGTTGGATGGTATTGCTCGACCATGGCTG | ACGTTGGATGTGCCATGTCTGTAGATGGAG | ACACCGCTGGGAAGTGT |
| tf-22 | ACGTTGGATGGTTTTACCCCTTTACACTAG | ACGTTGGATGCTGGCGACCATAATCTCTTG | TCATTCTGCAGTACTGCAGGAA |
| Gene |  |  |  |
| *Tf1* | ATGCCCTGAAGCTTAGGCAA | ACGCTGGAACTGTGAGGTGG |  |
| *Tf2* | GTGACACCTGGAGTGCCGAAAC | GTCACAGCTTCCAGTGCGATTA |  |
| Omp | CGAGGACTCCACTCCAGAGGACT | CAGTGCGGTGGTGTAAGTGG |  |
