# Supplementary material for: Polymorphism, selection and tandem duplication of transferrin genes in Atlantic cod (Gadus morhua) - Conserved synteny between fish monolobal and tetrapod bilobal transferrin loci
Source: BMC Genet. 2011 May 25;12:51. doi: 10.1186/1471-2156-12-51 (PMC3125230; doi:10.1186/1471-2156-12-51)
Supplement: Additional file 6 — Table S4 Accesion numbers of the proteins included in the phylogenetic analysis. Several distinct transferrin genes have been described in some teleost species. [file 1471-2156-12-51-S6.DOC]

| **Protein** | **Species** | **Accession number** |
| --- | --- | --- |
| Tf | *Melanogrammus aeglefinus* | CAC19019 |
| Tf | *Chionodraco rastrospinosus* | CAL92186 |
| Tf | *Chaenocephalus aceratus* | CAL92187 |
| Tf | *Notothenia coriiceps* | CAL92189 |
| Tf | *Oryzias latipes* | NP_001116384 |
| Tf | *Oreochromis niloticus* | ABB70391 |
| Tf | *Pagrus major* | AAP94279 |
| Tf | *Sparus aurata* | AEA41139 |
| Tf | *Gasterosteus aculeatus* | ENSGACP00000013229 |
| Tf | *Dicentrarchus labrax* | ACN80997 |
| Tf | *Larimichthys crocea* | CAM96032 |
| Tf1 | *Paralichthys olivaceus* | AAF33233 |
| Tf2 | *Paralichthys olivaceus* | AAF33234 |
| Tf1 | *Salmo marmoratus* | ACC55223 |
| Tf2 | *Salmo marmoratus* | ACC55224 |
| Tf1 | *Salmo trutta* | ACC55225 |
| Tf2 | *Salmo trutta* | BAA84102 |
| Tf1A | *Cyprinus carpio* | AAL57604 |
| Tf1F | *Cyprinus carpio* | ACD99641 |
| Tf1C | *Cyprinus carpio* | ACD99639 |
| Tf1D | *Cyprinus carpio* | AAM90970 |
| Tf | *Danio rerio* | NP_001015057 |
| Tf | *Ictalurus punctatus* | ACN42672 |
| Tf | *Homo sapiens* | AAB22049 |
| Tf | *Ciona intestinalis* | JGI ID 241714 |
| MTf | *Tetraodon nigroviridis* | CAF95005 |
| MTf | *Gasterosteus aculeatus* | ENSGACP00000008232 |
| MTf | *Oryzias latipes* | ENSORLP00000014067 |
| MTf | *Danio rerio* | XP_694299 |
| MTf | *Homo sapiens* | AAA59992 |
| nicaTf | *Ciona intestinalis* | JGI ID 241749 |
| OMP | *Tetraodon nigroviridis* | [CAG10345.1](http://www.ncbi.nlm.nih.gov/protein/47229931?report=genbank&log$=prottop&blast_rank=4&RID=XM6WJBPV016) |
| OMP | *Gasterosteus aculeatus* | ENSGACP00000013229 |
| OMP | *Oncorhynchus mykiss* | [NP_001117664.1](http://www.ncbi.nlm.nih.gov/protein/185134407?report=genbank&log$=prottop&blast_rank=3&RID=XM6WJBPV016) |
| OMP | *Danio rerio* | [AAH76449.1](http://www.ncbi.nlm.nih.gov/protein/49900517?report=genbank&log$=prottop&blast_rank=2&RID=XM6WJBPV016) |
