# Supplementary material for: Polymorphism, selection and tandem duplication of transferrin genes in Atlantic cod (Gadus morhua) - Conserved synteny between fish monolobal and tetrapod bilobal transferrin loci
Source: BMC Genet. 2011 May 25;12:51. doi: 10.1186/1471-2156-12-51 (PMC3125230; doi:10.1186/1471-2156-12-51)
Supplement: Supplementary file 6 [file 1471-2156-12-51-S4.DOC]

**tf-6**	Baltic Öland	Baltic Bornholm	Kattegat	North Sea	Molde	Malangen	Faroe Bank	Faroe Plateau	Båts-fjord	Greenland Nuuk	Greenland Sisimiut	Labrador	Nova Scotia	Georges bank
Baltic Öland						*	*			***	***	***	***	***
Baltic Bornholm	-0,001									***	***	***	***	***
Kattegat	0,000	-0,017								***	***	***	***	***
North Sea	0,036	0,002	0,001							**	***	***	***	***
Molde	0,042	-0,016	-0,017	-0,028							**	**	**	**
Malangen	0,123	0,067	0,064	0,005	-0,001						*	*	*	**
Faroe Bank	0,044	0,017	0,016	-0,010	-0,018	-0,006				*	***	**	***	***
Faroe Plateau	0,028	0,003	0,002	-0,013	-0,025	0,011	-0,006			**	***	***	***	***
Båtsfjord	0,184	0,082	0,078	-0,006	-0,011	-0,040	-0,019	0,001						*
Greenland Nuuk	0,237	0,194	0,190	0,115	0,097	0,028	0,100	0,139	0,015					
Greenland Sisimiut	0,352	0,314	0,309	0,233	0,200	0,120	0,228	0,272	0,102	0,018				
Labrador	0,503	0,440	0,433	0,316	0,265	0,157	0,300	0,363	0,131	0,025	-0,015			
Nova Scotia	0,419	0,373	0,367	0,278	0,235	0,145	0,272	0,323	0,124	0,028	-0,011	-0,017		
Georges bank	0,418	0,378	0,372	0,291	0,248	0,163	0,288	0,336	0,142	0,042	-0,006	-0,014	-0,010	

**tf-8**	Baltic Öland	Baltic Bornholm	Kattegat	North Sea	Molde	Malangen	Faroe Bank	Faroe Plateau	Båts-fjord	Greenland Nuuk	Greenland Sisimiut	Labrador	Nova Scotia	Georges bank
Baltic Öland						*			**	***	***	***	***	***
Baltic Bornholm	-0,001								*	***	***	***	***	***
Kattegat	0,000	-0,017							*	**	***	***	***	***
North Sea	0,036	0,002	0,001							*	***	***	***	***
Molde	0,042	-0,016	-0,017	-0,028							***	***	***	***
Malangen	0,123	0,067	0,064	0,005	-0,001						**	***	***	***
Faroe Bank	0,044	0,017	0,016	-0,010	-0,018	-0,006				*	***	***	***	***
Faroe Plateau	0,028	0,003	0,002	-0,013	-0,025	0,011	-0,006		*	**	***	***	***	***
Båtsfjord	0,297	0,211	0,205	0,089	0,060	-0,013	0,065	0,113				**	***	**
Greenland Nuuk	0,223	0,181	0,177	0,105	0,089	0,022	0,090	0,126	-0,032			***	***	***
Greenland Sisimiut	0,409	0,374	0,369	0,295	0,249	0,170	0,297	0,342	0,052	0,058		*	*	
Labrador	0,701	0,671	0,666	0,598	0,535	0,461	0,606	0,647	0,309	0,315	0,104			
Nova Scotia	0,669	0,642	0,637	0,577	0,518	0,451	0,589	0,626	0,308	0,313	0,107	-0,031		
Georges bank	0,610	0,582	0,576	0,512	0,452	0,381	0,525	0,564	0,238	0,245	0,058	-0,021	-0,019	

**tf-10**	Baltic Öland	Baltic Bornholm	Kattegat	North Sea	Molde	Malangen	Faroe Bank	Faroe Plateau	Båts-fjord	Greenland Nuuk	Greenland Sisimiut	Labrador	Nova Scotia	Georges bank
Baltic Öland						*	*		**	***	***	***	***	***
Baltic Bornholm	-0,001								*	***	***	***	***	***
Kattegat	0,000	-0,017							*	***	***	***	***	***
North Sea	0,036	0,002	0,001							**	***	***	***	***
Molde	0,042	-0,016	-0,017	-0,028						*	**	***	***	***
Malangen	0,123	0,067	0,064	0,005	-0,001						**	***	***	***
Faroe Bank	0,044	0,017	0,016	-0,010	-0,018	-0,006				**	***	***	***	***
Faroe Plateau	0,028	0,003	0,002	-0,013	-0,025	0,011	-0,006		*	**	***	***	***	***
Båtsfjord	0,297	0,211	0,205	0,089	0,060	-0,013	0,065	0,113				***	***	**
Greenland Nuuk	0,259	0,217	0,213	0,137	0,116	0,043	0,123	0,164	-0,025			***	***	***
Greenland Sisimiut	0,390	0,352	0,347	0,271	0,228	0,148	0,270	0,316	0,036	0,021		**	**	*
Labrador	0,726	0,697	0,692	0,627	0,568	0,494	0,633	0,673	0,346	0,306	0,157			
Nova Scotia	0,681	0,654	0,649	0,588	0,530	0,462	0,599	0,636	0,318	0,283	0,139	-0,028		
Georges bank	0,631	0,603	0,598	0,535	0,476	0,406	0,547	0,586	0,264	0,230	0,096	-0,016	-0,020	

**tf-11**	Baltic	Baltic	Kattegat	North	Molde	Malangen	Faroe	Faroe	Båts-	Greenland	Greenland	Labrador	Nova	Georges
	Öland	Bornholm		Sea			Bank	Plateau	fjord	Nuuk	Sisimiut		Scotia	bank
Baltic Oland							*		*	***	***	***	***	***
Baltic Bornholm	-0,012								*	**	***	***	***	***
Kattegat	0,032	0,009								*	**	***	***	***
North Sea	0,066	0,038	-0,009								*	***	***	***
Molde	-0,017	-0,029	-0,014	0,009						*	**	***	***	***
Malangen	0,064	0,030	-0,017	-0,020	-0,001						*	***	***	***
Faroe Bank	0,050	0,029	-0,009	-0,012	0,005	-0,017					**	***	***	***
Faroe Plateau	0,025	0,007	-0,013	-0,004	-0,013	-0,012	-0,004			*	**	***	***	***
Båtsfjord	0,205	0,144	0,025	-0,010	0,060	-0,013	0,004	0,043				***	***	**
Greenland Nuuk	0,196	0,161	0,069	0,032	0,101	0,032	0,047	0,089	-0,034			***	***	***
Greenland Sisimiut	0,275	0,240	0,137	0,088	0,161	0,085	0,114	0,167	-0,013	-0,008		***	***	**
Labrador	0,742	0,716	0,621	0,564	0,628	0,557	0,601	0,659	0,414	0,382	0,285			
Nova Scotia	0,720	0,697	0,611	0,558	0,617	0,551	0,595	0,648	0,419	0,387	0,292	-0,030		
Georges bank	0,640	0,614	0,517	0,459	0,518	0,448	0,502	0,562	0,304	0,279	0,188	-0,011	-0,007	
**tf-13**	Baltic	Baltic	Kattegat	North	Molde	Malangen	Faroe	Faroe	Båts-	Greenland	Greenland	Labrador	Nova	Georges
	Öland	Bornholm		Sea			Bank	Plateau	fjord	Nuuk	Sisimiut		Scotia	bank
Baltic Öland						*	*		*	**	***	***	***	***
Baltic Bornholm	-0,001								*	*	***	***	***	***
Kattegat	0,000	-0,017							*	*	***	***	***	***
North Sea	0,036	0,002	0,001								***	***	***	***
Molde	0,042	-0,016	-0,017	-0,028							**	**	**	**
Malangen	0,123	0,067	0,064	0,005	-0,001						*	**	*	*
Faroe Bank	0,044	0,017	0,016	-0,010	-0,018	-0,006					***	***	***	***
Faroe Plateau	0,021	-0,003	-0,004	-0,012	-0,026	0,024	-0,002			*	***	***	***	***
Båtsfjord	0,236	0,143	0,138	0,036	0,028	-0,029	0,014	0,072						
Greenland Nuuk	0,155	0,109	0,105	0,040	0,032	-0,015	0,024	0,068	-0,032		*	*	*	*
Greenland Sisimiut	0,352	0,314	0,309	0,233	0,200	0,120	0,228	0,295	0,066	0,078				
Labrador	0,447	0,405	0,399	0,314	0,268	0,179	0,311	0,387	0,114	0,130	-0,004			
Nova Scotia	0,373	0,336	0,331	0,256	0,219	0,139	0,252	0,319	0,082	0,095	-0,009	-0,007		
Georges bank	0,352	0,314	0,309	0,233	0,200	0,120	0,228	0,295	0,066	0,078	-0,010	-0,004	-0,009	
**tf-22**	Baltic	Baltic	Kattegat	North	Molde	Malangen	Faroe	Faroe	Båts-	Greenland	Greenland	Labrador	Nova	Georges
	Öland	Bornholm		Sea			Bank	Plateau	fjord	Nuuk	Sisimiut		Scotia	bank
Baltic Öland						*	*		**	***	***	***	***	***
Baltic Bornholm	-0,001								*	**	***	***	***	***
Kattegat	0,000	-0,017							*	**	***	***	***	***
North Sea	0,036	0,002	0,001							*	***	***	***	***
Molde	0,042	-0,016	-0,017	-0,028							**	***	***	***
Malangen	0,123	0,067	0,064	0,005	-0,001						**	***	***	**
Faroe Bank	0,044	0,017	0,016	-0,010	-0,018	-0,006				*	***	***	***	***
Faroe Plateau	0,028	0,003	0,002	-0,013	-0,025	0,011	-0,006		*	*	***	***	***	***
Båtsfjord	0,297	0,211	0,205	0,089	0,060	-0,013	0,065	0,113				*	**	
Greenland Nuuk	0,218	0,171	0,167	0,092	0,077	0,011	0,074	0,111	-0,037		*	**	***	
Greenland Sisimiut	0,409	0,374	0,369	0,295	0,249	0,170	0,297	0,342	0,052	0,069				
Labrador	0,550	0,513	0,507	0,428	0,365	0,283	0,435	0,483	0,138	0,160	0,000			
Nova Scotia	0,569	0,540	0,534	0,468	0,407	0,335	0,481	0,521	0,192	0,216	0,030	-0,018		
Georges bank	0,409	0,374	0,369	0,295	0,249	0,170	0,297	0,342	0,052	0,069	-0,017	0,000	0,030	
**All six SNP-loci**	Baltic	Baltic	Kattegat	North	Molde	Malangen	Faroe	Faroe	Båts-	Greenland	Greenland	Labrador	Nova	Georges
	Öland	Bornholm		Sea			Bank	Plateau	fjord	Nuuk	Sisimiut		Scotia	bank
Baltic Öland														
Baltic Bornholm	-0,005													
Kattegat	0,017	-0,007												
North Sea	0,046	0,013	-0,003											
Molde	0,026	-0,019	-0,016	-0,018										
Malangen	0,112	0,060	0,044	-0,001	-0,001									
Faroe Bank	0,045	0,020	0,009	-0,011	-0,013	-0,009								
Faroe Plateau	0,026	0,003	-0,003	-0,011	-0,022	0,008	-0,005							
Båtsfjord	0,259	0,175	0,137	0,047	0,048	-0,019	0,034	0,079						
Greenland Nuuk	0,219	0,176	0,153	0,087	0,089	0,022	0,078	0,119	-0,025					
Greenland Sisimiut	0,368	0,332	0,311	0,239	0,217	0,138	0,242	0,293	0,049	0,039				
Labrador	0,651	0,616	0,589	0,515	0,478	0,397	0,528	0,582	0,264	0,249	0,102			
Nova Scotia	0,613	0,583	0,559	0,492	0,458	0,384	0,509	0,557	0,261	0,246	0,103	-0,022		
Georges bank	0,542	0,510	0,485	0,415	0,384	0,307	0,431	0,481	0,191	0,173	0,056	-0,011	-0,005	
